# Immediate implant placement in anterior teeth with grafting material of autogenous tooth bone vs xenogenic bone

**DOI:** 10.1186/s12903-019-0970-7

**Published:** 2019-12-02

**Authors:** Dong Wu, Lin Zhou, Jichao Lin, Jiang Chen, Wenxiu Huang, Yonghui Chen

**Affiliations:** 1 Department of Oral Implantology, Affiliated Stomatological Hospital of Fujian Medical University, Fuzhou, 350002 Fujian China; 2 Department of Stomatology, Xiamen branch, Zhongshan hospital, Fudan university, Xiamen, 361000 Fujian China; 3 Department of Stomatology, Zhangzhou Affiliated Hospital of Fujian Medical University, Zhangzhou, 363000 Fujian China

**Keywords:** Autogenous tooth, Bone graft, Immediate implant placement, Implant dentistry

## Abstract

**Background:**

The aim of the study was to compare the efficacy of the autogenous tooth bone and xenogenic bone grafted in immediate implant placement with bone defect.

**Methods:**

Thirty patients whose compromised anterior teeth need immediate implant placement were enrolled. Autogenous tooth bone made from the extracted teeth by chair-side or the xenogenic bone were used to repaired bone defect. Clinical examination, radiographic assessment about the horizontal bone change in the level of 0 mm, 3 mm and 6 mm below the implant neck and the marginal bone loss were made immediately, 6 and 12 months after implant placement. Questionnaire of the feelings about the surgery were made at the time of removing the sutures.

**Results:**

All implants achieved the success criteria without any complications at the follow-up period. The percent of the horizontal bone change and the marginal bone loss at 6 and 12 months were almost the same between two groups (*P* > .05). The horizontal bone loss at the first or the latter 6 months was almost the same (*P* > .05). But the horizontal bone loss at the 6 mm level was less than the 0 mm and 3 mm levels at 6 and 12 months (*P* < .05). Meanwhile patients seem more satisfied with the autogenous tooth bone derived from the questionnaire.

**Conclusion:**

The bone volume change in the facial part of the implant after immediate placement is almost the same between two groups. Providing clinical evidence that the autogenous tooth bone made from compromised tooth can be an acceptable bone graft material.

## Background

With the development of dental implant technology and biomaterials, dental implants have become the preferred treatment for dentition and edentulous patients. Within the development of decades, several strategies of implant placement had been developed. Among them, the immediate implant placement in anterior teeth had been widely accepted after it been introduced in the late 1970s by Schulte and Heimke [1], because it can avoid the buccal bone resorption, shorten the period of treatment time, and avoid the lack of teeth due to the provisional restoration [2].

Bone deficiency happened among in over half of implantation sites [3]. Meanwhile, there are half of immediate implant placement in anterior teeth need bone augmentation, due to the defect of buccal wall which may cause by trauma, apical periodontitis and periodontitis [4]. Bone augmentation cannot be carried out without bone graft materials. Autogenous bone, allogenic bone, xenogenic bone, and alloplastic materials are bone graft materials that are presently used in dental clinic. Autogenous bone graft material is considered as the golden standard since it’s capable of osteogenesis, osteoinduction, and osteoconduction. However, the generation of donor area which cause secondary defect, more trauma and complexed, and the limited harvest amount of collected bone restricted its application [5]. Allogenic bone and xenogenic bone may lead to infection or immune rejection. Limited osteogenic effect, high cost of treatment and low degree of patient’s acceptance restrict their clinical application [6].

In recent years, some cases reported that autogenous tooth bone graft material made from compromised teeth was applied in bone deficient areas and achieved a good clinical efficacy [7–11]. Especially in the case of immediate extraction implant placement, the compromised teeth can be used as a bone graft material which is more accepted by patients. Autogenous tooth bone graft material was first development in 2008 and used as a bone graft material in guided bone regeneration [12]. As we know that the components of tooth are very similar to the alveolar bone. The total inorganic content, organic content and water of the enamel and dentin is 95, 0.6, 4 and 70%, 20, 10% respectively, which is similar to those contents of 65, 25, 10% in alveolar bone [13]. The histological outcomes of the discarded teeth after demineralization are similar to autogenous bone grafts, which make it be the perfect bone graft materials for its osteoconductive and osteoinductive property [8]. Thought the clinical outcomes of the autogenous tooth bone graft materials had been widely reported, but the comparison with other bone graft materials is still rare.

The aim of our present retrospective study was to compare the clinical effect of the autogenous tooth bone graft materials with the xenogenic bone graft materials (Bio-Oss) in bone regeneration of immediate extraction implant placement with a defect of labial bone wall.

## Methods

### Patient selection

This was a retrospective observational study of patients, whose anterior teeth need to be extracted with a defect of labial bone, need immediate implant placement with bone augmentation using bone graft material. From March 2016 to May 2017, 30 patients (12 women; 18 men), with a mean age of 48 ± 16.7 years (range 19 to 67 years) were collected from department of oral and maxillofacial implant research center, Affiliated Stomatological Hospital of Fujian Medical University. All patients were informed about the surgical and restoration treatment procedure. The study design was performed in accordance with the Helsinki Declaration (revised in 2008).

The inclusion criteria were as follow: (1) Anterior teeth need to be extracted with a defect of labial bone (horizontal or vertical bone defect). (2) the teeth without acute inflammation. (3) Without uncontrolled systemic disease which is not suitable for implantation. (4) Good systemic and oral health. And the exclusion criteria were: (1) Heavy smoker (> 10 cigarettes/day). (2) Acute inflammation in the site of implantation and adjacent tissue. (3) A history of radiotherapy in the head or neck region. (4) With systemic disease like uncontrolled diabetes mellitus, coagulation disorders, alcohol or drug abuse not suitable for implantation.

### Preoperative work-up

All patients perform general oral examination and CBCT examination to observe the structure of the teeth which need to be extracted and the labial bone before surgery. Then we measured the available bone width and bone height to determine the treatment program and reach consensus with patients. All patients got the serial of professional oral hygiene with scaling and root planning 2 weeks before surgery. For the prevention of infection and better plaque control, all patients were given antibiotic 3 days before surgery and mouthrinsed with 0.2% chlorhexidine 1 week before surgery. At the same time, surgeon measured the CBCT to precisely assess the width, the depth and the defect of the labial bone, in order to perform better implant placement and choose the most appropriate implant.

### Autogenous tooth bone graft preparation

**Fig. 1:**
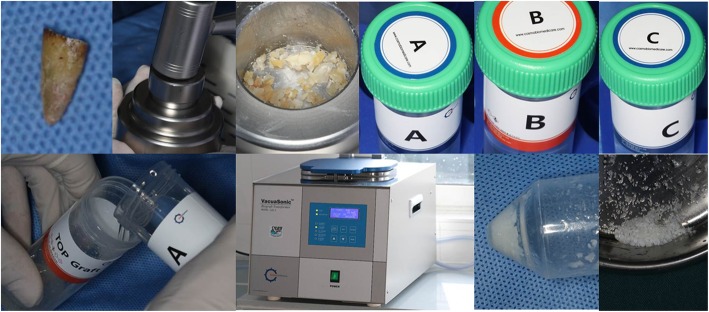
Chairside preparation of autogenous tooth bone graft materials. Autogenous tooth without retention value was removed with minimally invasive extraction. Surface preparation and pretreatment were performed on the teeth. The tooth was crushed into debris. Bone powder was prepared. Demineralization, sterilization and rinse were performed for autogenous tooth bone graft materials

All the preparation of autogenous tooth bone graft material was done by the same dentist who was well skilled with this technology. Autogenous tooth bone graft material derived from tooth which need to be extracted without the retention value, and prepared following the instructions of vacuum ultrasonic autoclaved bone preparation equipment (VacuaSonic®, Korea). Autogenous teeth without retention value were extracted with a minimally invasive tooth extraction device, under routine local infiltration anesthesia or block anesthesia with Primacaine® (4% Articaine,1/100000 adrenaline, ACTEON) 30mins before surgery. The residual periodontal ligament on root surfaces was removed. Caries and restorations were removed by the grinding needle. Then the tooth was crushed into debris by a hammer in an iron container, meanwhile the dental pulp or canal filling material was removed. The size of the debris is determined by a sieve. At last the bone debris was put into a vacuum ultrasonic autoclaved bone preparation equipment with different solution according to the manufacture. After demineralization, peracetic acid sterilization and rinse, autogenous tooth bone graft material was prepared eventually as it showed in the Fig. 1.

### Surgical and prosthetic Proceduce

**Fig. 2:**
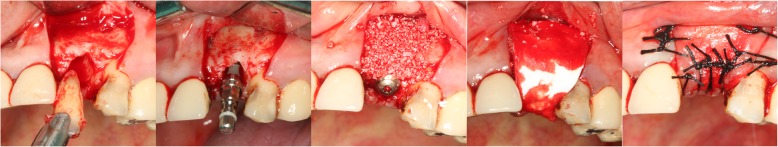
Surgical proceduce of the implant placement and bone grafting

All the surgeries were performed by the same surgeon with more than 25 years of experience. Tooth extraction was made under local anesthesia with Primacaine® (4% Articaine,1/100000 adrenaline, ACTEON) before surgery. Then the full-thickness midcrestal incision and vertical releasing incision in distal side were made, vertical releasing incision in mistal side was made if necessary. The extraction socket and the labial bone defect were exposed by buccal and palatal flap reflection. Inflammatory granulation tissue was removed before hole preparation. The implant position was marked on the palatal bone wall of the extraction socket with a small round drill (diameter of 2.0 mm). Subsequently, pioneer drill was used to make the right implant depth which was 3-4 mm below the gingival level of final restoration and implant position which was at the centerperdetermined mesiodistal width with a minimal distance of 2 mm from the adjacent tooth and a few palatal side of the buccal and palatal aspect. Then the drills were used to expand the hole to the final size step by step, and insert the screw type implant with the cover screw placed. The autogenous tooth bone (atuoBT) or the xenogenic bone (xenoB, Geistlich Bio-Oss) were used to filled the gap between the facial bone wall and the implant and the defect of the facial bone to reach the enough buccal bone supported, and then the graft materials were covered with absorbable barrier membranes(Bio-Gide, Geistlich). Finally, the flap was repositioned and sutured. The brief process of the surgery is showed in Fig. 2. According to implant routine postoperative medical advice, a certain amount of antibiotics were given to prevent wound infection and excessive bleeding. After 4 mouths healing, the prosthetic procedure was performed with a titanium abutment and a zirconium dioxide crown. Patients underwent CBCT examination immediately, 6 months and 1 year after surgery.

### Outcome measurements

#### Implant success

The implant success criteria in our study was based on the criteria of Albrektsson, Zarb, Worthington and Eriksson(1986) and of Buser, Weber and Lang(1990). The following are the criteria of the implant success: the absence of mobility, the absence of acute or chronic peri-implant infection, the absence of radiolucency around the implant, without pocket probing depth (PPD) ≧ 5 mm, and without vertical bone loss ≧ 1.5 mm in the first year. The cases will be defined as failure if it can’t reach any one of the success criteria.

### Clinical assessment

Swelling, wound dehiscence and other adverse events were observed at 3 days and 7 days after implant placement. The guided bone regeneration using different graft materials regard as failure when the following clinical feature arisen: emerging of the fistula, the particle of the bone graft material flow out from the fistula or mucosal dehiscence, and the chronic inflammation.

### Radiographic assessment

**Fig. 3:**
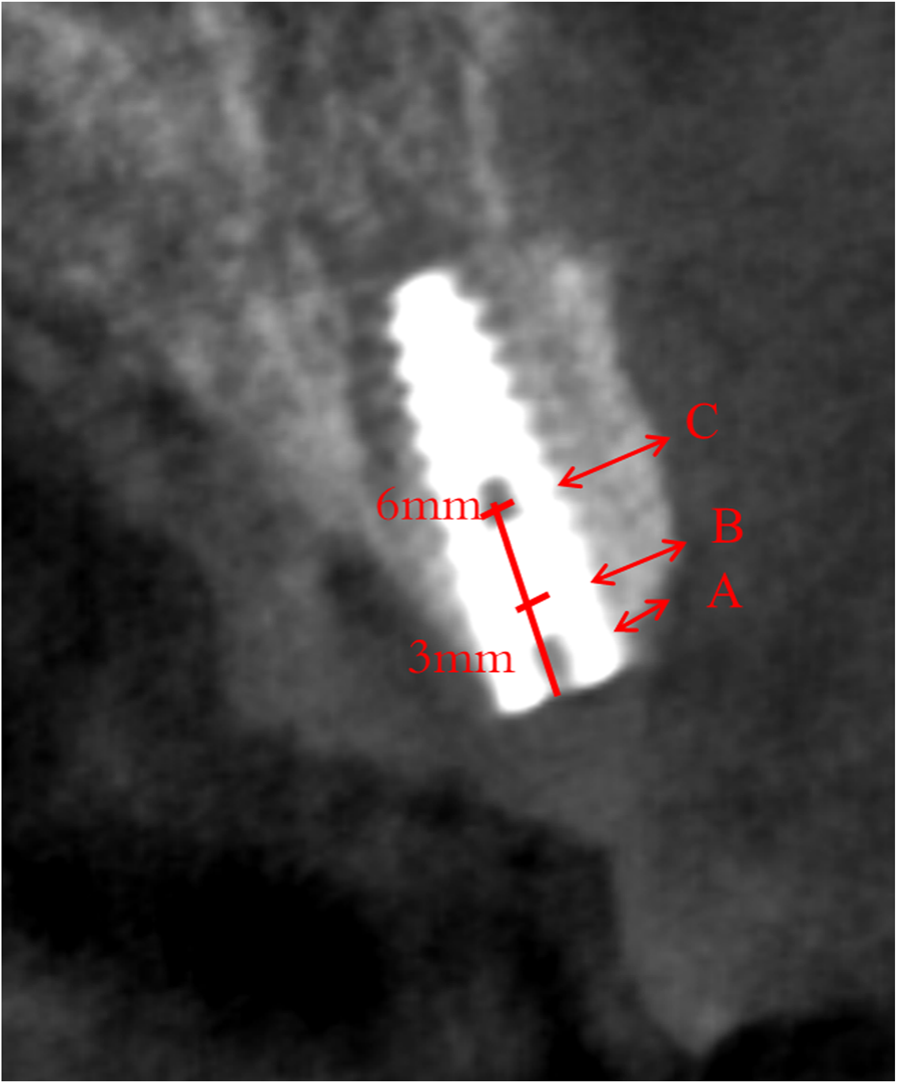
The measured point of facial bone. **a** represent the bone width at the level of implant neck or the top of the buccal bone. **b** and **c** represent the bone width of the 3 mm and 6 mm below the implant neck level

All the patients were measured by CBCT scanning before implant placement, immediately after implant placement, 6 months and 12 months follow-up. And all the measurements were done by one dentist who did not know which group was. Marginal bone level and horizontal bone change at the facial side of implant were measured. The centre of the implant was set as a vertical reference line in the CBCT image and all the measured points were perpendicular to it. We measured the labial horizontal bone width perpendicular to the vertical line of the implant surface at the implant neck level or the top of the buccal bone (which is regarded as 0 mm), 3 mm and 6 mm apical to the implant neck level as it shown in Fig. 3. The stability of the labial horizontal bone was evaluated by the percent of the horizontal bone loss. And the percent of the horizontal bone loss at each time point and each measured point were calculated by the following formula: (width of base line – width of following-up time)/ width of base line. Meanwhile the marginal bone level (MBL) at the implant neck level was measured at different following-up time according to the CBCT image.

### Patient satisfaction evaluation

Patient satisfaction was evaluated by a questionnaire based on the Visual Analog Scales (VAS 0–10), which is focused on the pain, swelling, satisfaction of the surgery process. All patients answer the questionnaire at the time of removing the suture. The questionnaire was show in Additional file 1.

### Statistical analysis

The data about the percent of the horizontal bone loss, marginal bone level at different following-up time and between different bone graft materials were compared with each other using the Independent-Samples t-test with the SPSS 22.0 software. So did the results of the questionnaire. The percent of the horizontal bone loss at different measure level were compared with each other using one-way ANOVA. *P* values < 0.05 were defined as statistically significant.

## Results

### Basic information of patients

In our study, the total of 30 patients were included (12women/ 18men, mean age: 40.43 ± 13.58 years). Among the 30 implants, 15 belonged to the group of autogenous tooth bone graft and the rest belonged to the group of xenogenic bone graft. The implant location and the basic information of the patients were detailed in the Table 1.

**Table 1:** Basic information of the involved patients

Patients	Autogenous tooth bone			Xenogenic bone (Bio-Oss)		
No.	Age Ranges	Tooth extraction site	Implant brand and sizes (mm)	Age Ranges	Tooth extraction site	Implant brand and sizes (mm)
1	60–70	12	Straumann 3.3*13	20–30	12	BEGO 3.25*13
2	40–50	21	BEGO 4.1*13	30–40	21	BEGO 3.25*13
3	20–30	22	BEGO 3.25*11.5	30–40	22	BEGO 3.25*13
4	20–30	12	BEGO 3.25*13	20–30	12	Straumann 3.3*13
5	30–40	21	BEGO 3.75*13	50–60	21	BEGO 3.75*13
6	60–70	12	Straumann 3.3*13	40–50	21	Straumann 3.3*13
7	20–30	12	Straumann 3.3*13	50–60	22	BEGO 3.25*13
8	10–20	13	BEGO 4.1*13	50–60	23	BEGO 3.75*13
9	30–40	12	BEGO 3.25*11.5	50–60	11	Straumann 3.3*13
10	40–50	21	Straumann 3.3*13	30–40	22	Straumann 3.3*13
11	40–50	21	BEGO 3.75*15	40–50	11	BEGO 3.75*13
12	30–40	13	MIS 3.75*13	30–40	21	BEGO 3.25*13
13	50–60	22	BEGO 3.25*13	60–70	21	BEGO 3.75*11.5
14	40–50	23	MIS 3.75*13	50–60	11	BEGO 3.75*13
15	20–30	21	BEGO 3.75*13	30–40	11	BEGO 3.75*13

### Implant success

All the implant in the both group reach the success criteria of our paper mentioned above during the entire observation period. Neither the implants nor the bone graft materials had any biological and mechanical complications, like peri-implantitis and infection during the follow-up period.

### Clinical observation

We observed whether the pain, swelling and other complaint happened in the patient at the day 3 and 7 after surgery. Both two groups had different level of pain and swelling at the day 3 and 7 after surgery. But none of the 30 patients had the clinical manifestation of infection and wound dehiscence.

### Radiographic assessment

**Fig. 4:**
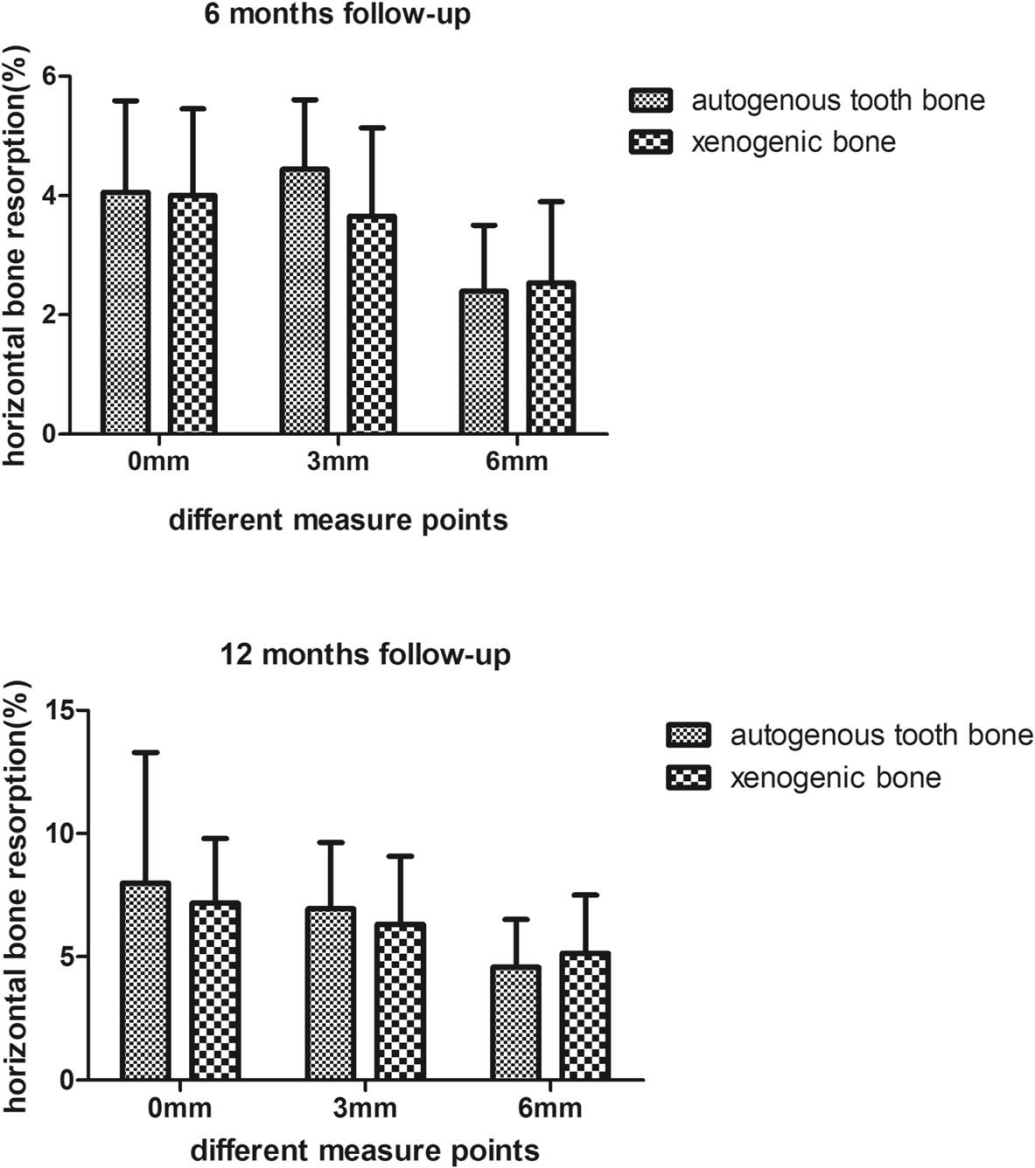
The change of the horizontal bone at 6 and 12 months in the level of 0 mm, 3 mm and 6 mm between the autogenous tooth bone and xenogenic bone

**Fig. 5:**
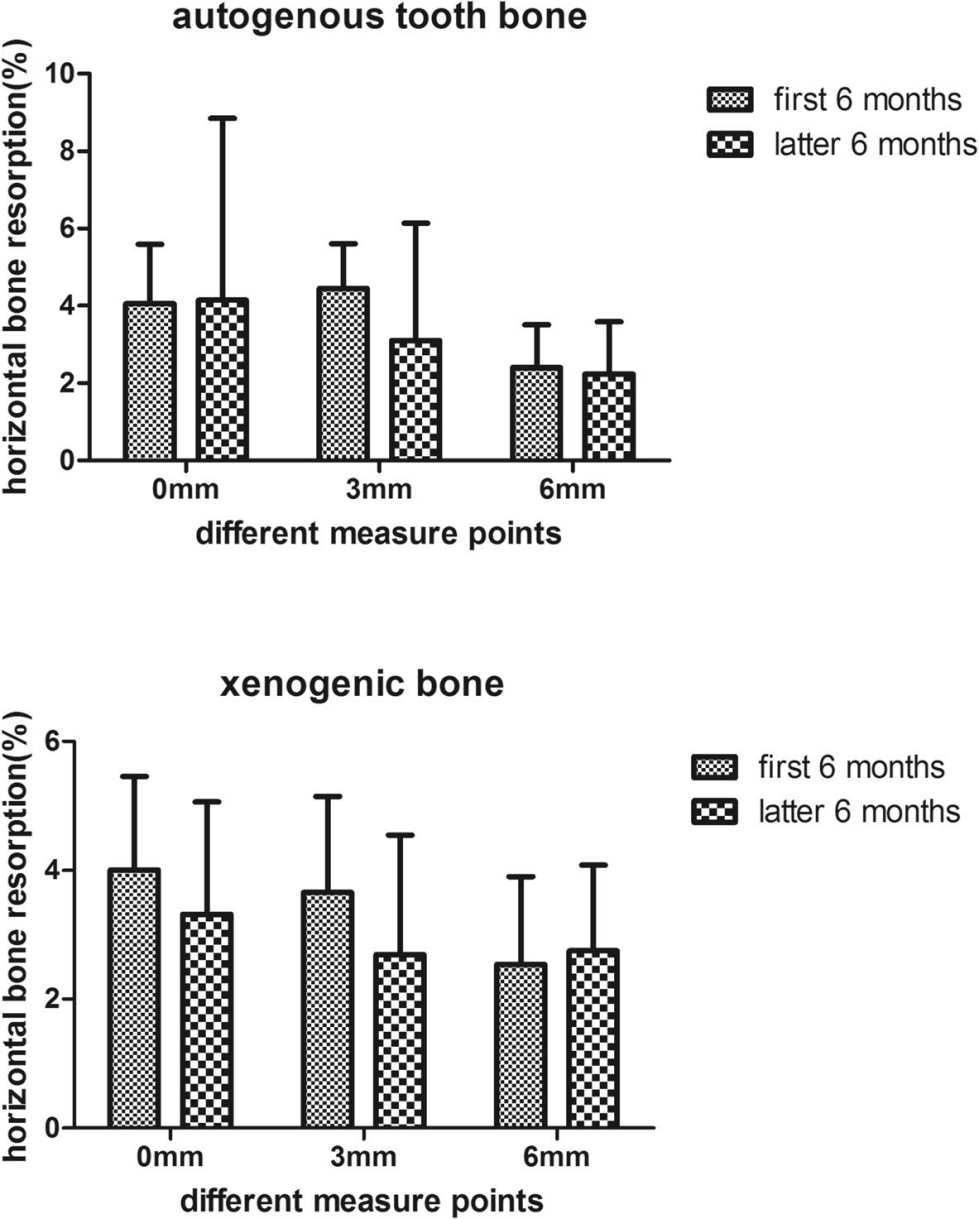
The change of the horizontal bone at the group of autogenous tooth bone and the xenogenic bone in the level of 0 mm, 3 mm and 6 mm between the first 6 months and latter 6 months

**Fig. 6:**
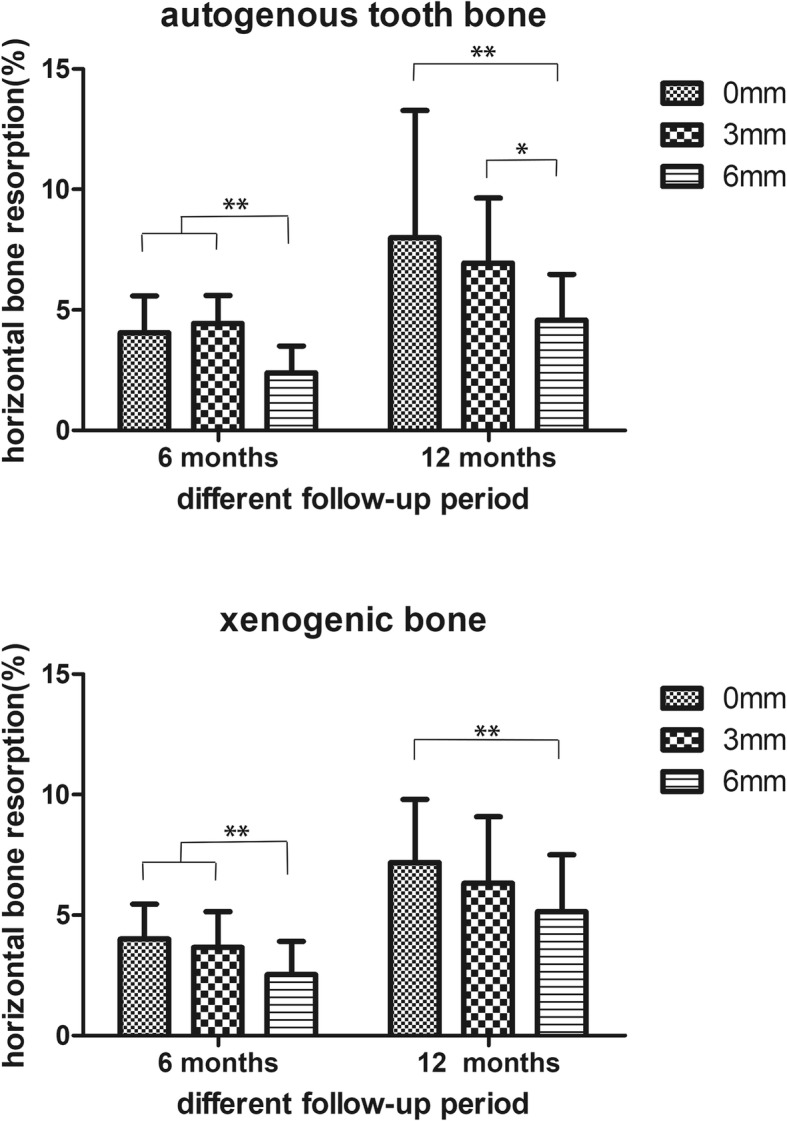
The change of the horizontal bone at 6 months and 12 months in the group of autoTB and xenoB between the level of 0 mm, 3 mm and 6 mm. ** represent *P* values < 0.05, * represent *P* values < 0.1

The width of the horizontal bone in the level of 0 mm, 3 mm and 6 mm at the 6 months and 12 months in the different groups were measured. And the percent of the horizontal bone change was calculated according to the formula above. The percent of the horizontal bone change at 6 months in the level of 0 mm, 3 mm and 6 mm were (4.06 ± 1.53), (4.45 ± 1.16) and (2.40 ± 1.11) in the group of autoBT, and (4.01 ± 1.45), (3.66 ± 1.49) and (2.54 ± 1.36) in the group of xenoB. And the percent of the horizontal bone change at 12 months in the level of 0 mm, 3 mm and 6 mm were (7.99 ± 5.29), (6.94 ± 2.70) and (4.58 ± 1.91) in the group of autoBT, and (7.18 ± 2.62), (6.31 ± 2.76) and (5.15 ± 2.36) in the group of xenoB. The change of the horizontal bone at 6 and 12 months in the level of 0 mm, 3 mm and 6 mm between the two groups had no significant different as it showed in the Fig.4. Meanwhile the change of the horizontal bone at the group of autogenous tooth bone and the xenogenic bone in the level of 0 mm, 3 mm and 6 mm between the first 6 months and latter 6 months also had no significant different as it showed in the Fig.5. The change of the horizontal bone at 6 months and 12 months in the group of autoBT at the level of 6 mm is significant lower than the level of 0 mm and 3 mm, but there was no different between the level of 0 mm and 3 mm. And the change of the horizontal bone at 6 months in the group of xenoB in the level of 6 mm was significant lower than the level of 0 mm and 3 mm, but only the 0 mm level was significant higher than the 6 mm. This result showed in the Fig.6.

**Fig. 7:**
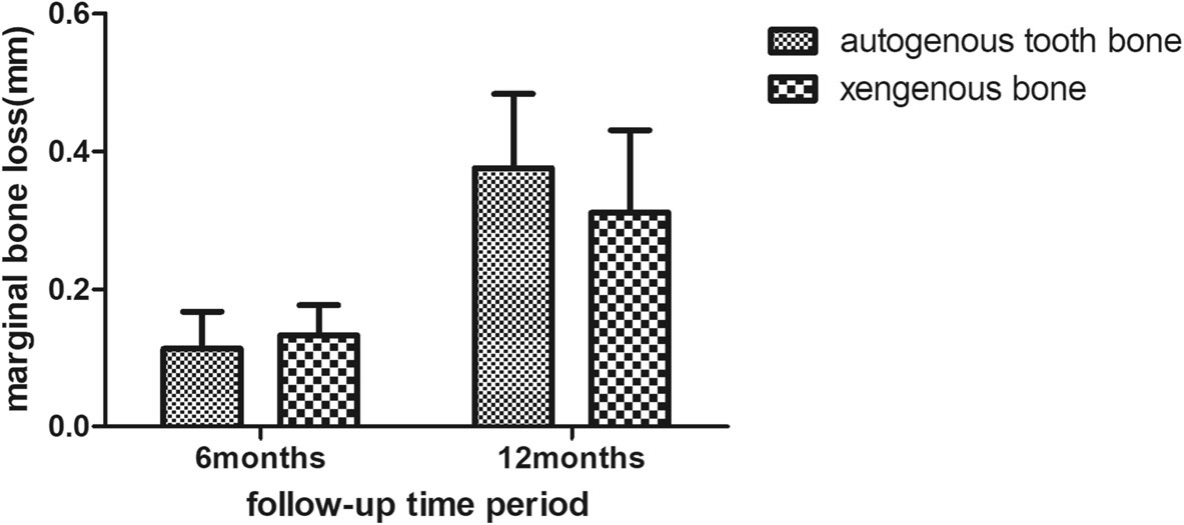
The marginal bone loss at 6 months and 12 months in the group of autoBT and xenoB

Meanwhile the marginal bone loss was measured by CBCT image at 6 and 12 months. The marginal bone loss at 6 months were (0.11 ± 0.05) mm and (0.13 ± 0.04) mm in the group of autoBT and xenoB respectively. And the marginal bone loss at 12 months were (0.38 ± 0.1) mm and (0.31 ± 0.12) mm in the group of autoBT and xenoB respectively. Both the different had no statistic significant as it showed in Fig. 7.

### Patient satisfaction

**Fig. 8:**
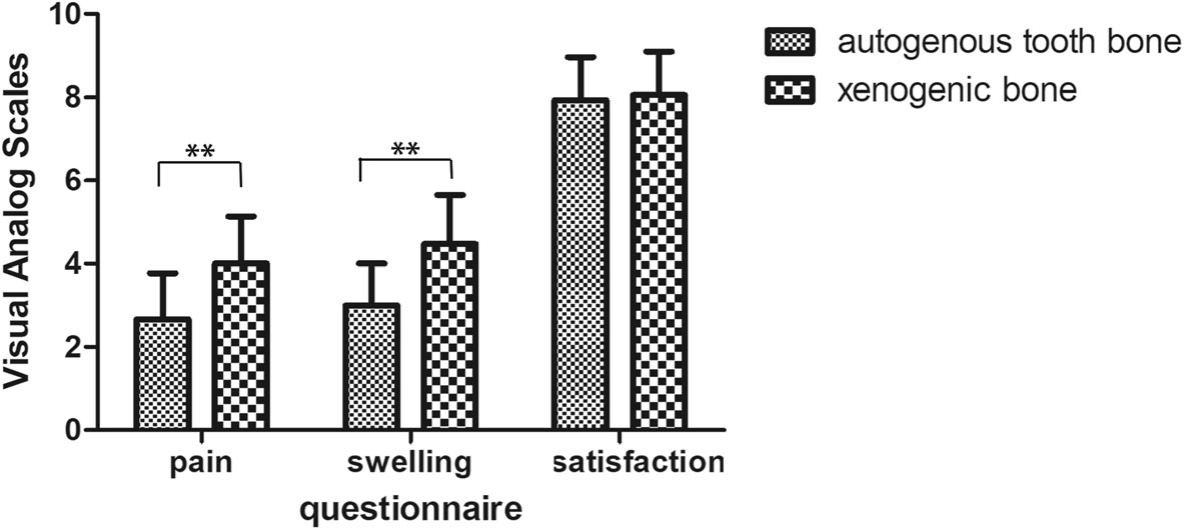
The value in pain, swelling and satisfaction in the group of autogenous tooth bone and xenogenic bone

All the patients answered the questionnaire at the time of removing the suture. The value of the pain, swelling and the satisfaction were (2.60 ± 1.12), (3.00 ± 1.00) and (7.87 ± 0.92) in the group of autogenous tooth bone, and (4.00 ± 1.13), (4.47 ± 1.19) and (8.07 ± 1.03) in the group of xenogenic bone respectively as it shows in the Table 2. The pain and the swelling in the group of autogenous tooth bone were lower than the group of xenogenic bone, but the satisfaction of the surgery process is almost the same. The value in pain and swelling showed statistically significant difference as the Fig. 8 shown.

**Table 2:** The questionnaire of patients with different bone graft materials

Questionnaire	Graft materials	Number	Mean	SD
Pain	Autogenous tooth bone	15	2.6000	1.12122
	Xenogenic bone	15	4.0000	1.13389
Swelling	Autogenous tooth bone	15	3.0000	1.00000
	Xenogenic bone	15	4.4667	1.18723
Satisfaction	Autogenous tooth bone	15	7.8667	0.91548
	Xenogenic bone	15	8.0667	1.03280

## Discussion

In the present retrospective study, the autogenous tooth bone graft which was made by the extracted tooth chairside were successful used in the immediate implant placement in the fresh socket with facial bone defect. The protocol of the immediate implant placement using different bone graft material had been well evaluated in some systematic reviews [2, 14]. And the protocol of immediate implant placement in fresh extraction socket may help to maintain the bone and soft tissue stable, enhancing the survival of the implant, shorten the treatment time and achieve patients’ satisfaction [15].

But in many cases, there was a defect in the facial bone of the compromised teeth in the esthetic zone, and the bone graft is needed after implant placement. In the present study, a total of 30 implant had been inserted into fresh socket with facial bone defect, half of it using autogenous tooth bone graft material and the rest of it using xenogenic bone (Bio-Oss). Neither the implant nor the graft material fail to reach the success criteria. The result consistent with other clinic studies. As patricia et al. reviewed that the mean implant survival rate was 97.7% while using the autogenous tooth bone as a graft material with a limited number of cases [13]. Also in other recent systematic review the implant survival rate using bone graft material were higher than 90% (range from 90 to 100%) [16, 17]. Meanwhile, many studies had shown that Autogenous tooth bone graft materials have been applied for lateral sinus floor elevation, guided bone regeneration, alveolar bone preservation and other bone augmentation surgery [18], and show high bone formation activity and excellent biocompatibility [19]. The chemical composition of the teeth, especially dentin, closely approximates bone tissue. Dentin is rich in bone morphogenetic protein (BMP) which can promote bone marrow mesenchymal stem cell differentiation and accelerating osteogenesis [20]. The dentin consists of low crystalline hydroxyapatite similar to bone tissue, compared to the high crystalline enamel of hydroxyapatite structure. Meanwhile, previous studies also showed that the enamel could not be easily degraded by osteoclasts because of its highly mineralized calcium phosphate crystals, resulting in the difficulty of osteogenesis factors release such as BMPs and the delay of BMSCs migration, adhesion and differentiation on the material surface [21]. Hence, the osteoinductivity and osteoconductivity of the enamel and dentin make the autogenous tooth bone a perfect bone graft material and high success rate.

We measured the different level of the implant buccal bone width at different follow-up time to observed the stability of the horizontal bone. As we can see from the Figs 4, 5 and 6 about the change of the horizontal bone, there is no statistic difference between the two bone graft materials in three different measured level at the 6 months and 12 months follow-up period, and there is no statistic difference between the first and latter 6 months follow-up at two different bone graft material in three different measured levels. These results indicated that the horizontal bone loss in the autogenous tooth bone group is almost the same with the xenogenic bone in three measured levels at the 12 months following up and the horizontal bone loss at first and latter 6 months is also the same. But the horizontal bone loss at the level of 6 mm was much more than the level of 0 mm and 3 mm at the different follow-up time and different bone graft material, while the level of 0 mm is the same with the level of 3 mm. We can drive from the result that the apical bone may be more stable than the marginal bone. All this percent of the horizontal bone loss was accepted, and was consensus with other research. Francesco et al. reported that the volumetric tissue changes after immediate extraction placement in the esthetic area can be minimized by a provisional restoration and bone graft inserted simultaneously with implant placement [22]. And Fabio’s research proved that the protocol of flap approach would affected the bone volume changes in the immediate implant placement, and the reduction of bone width is almost 10% in the first 6 month [23].

Marginal bone is significant important for the facial gingiva of an implant, and it is also an important clinical parameter for the implant long term success. Overall, the marginal bone loss in the two groups ranging from 0.02 to 0.59 which was accepted by the clinical. And the marginal bone loss in the immediate implant placement using autogenous tooth bone and xenogenic bone were almost the same, which means the marginal bone level or the gingiva level was stable in the two bone graft materials. Many other clinical studies also showed that the implant placement in the fresh socket with GBR had an acceptable marginal bone loss [24, 25]. The Eugenio et al. indicated that the mean marginal bone loss was 0.67 ± 0.40 mm (ranging from 0 mm to 1.6 mm**)** in the immediate implant placement with the follow-up of 4 years [26]. And some characteristics of the implant like tapered, platform-switch, laser-microtextured would affect the marginal bone level. Iorio-Siciliano V et al. reported that implants with a laser-microtextured collar can reduce the loss of marginal bone compared with tradition implant [27]. Iorio-Siciliano V et al. also reported that platform-switch can maintained marginal bone level [28]. There are 22 implants with the platform-switch in the 30 cases of the present study, which may help to reduce the loss of the marginal bone.

In the present study, the results of the questionnaire about the pain and swelling seems better in the group of the autogenous tooth bone graft material. This indicated that GBR with the autogenous tooth bone may cause less inflammatory reactive. Because the xenogenic bone grafted in the bone defect can be regard as a foreign substance insert into body, which can generate an immune and inflammatory reaction and it can be referred to as “osteoimmunology” [29, 30]. After the bone graft material inserted in the bone defect, the immune cell like macrophage will secrete pro-inflammatory cytokines as TNF-α, IF-1, which caused a transient abnormal enlargement of the operative region [31]. Autogenous tooth bone is consisted of demineralized dentin matrix (DDM) largely which is demineralized from dentin, a mainly structure of teeth [32]. The DDM which is the internal stuff may arouse less auto-immunity, therefore the less swelling and pain happened. This is the hypothesis based on the osteoimmunology, the underlying reason still need to be exposed by molecular mechanism research. The auto-suggestion of the patients about the graft bone is part of themselves may ben benefit the postoperative reactions.

### Limitations

The limitations of this study: the clinical observation period was 12 months which is not long enough to observe the long-term stability of the grafted bone. And the cases were done three or 4 years age, so the design the flap and the selection of the surgical consumables like the suture may be behindhand. The sample size of the research may not enough for a rigid statistical analysis. Thought the measurement was done by one dentist, the subjective bias was inevitable.

## Conclusion

1. Immediate implant placement in interior teeth with facial bone defect using autogenous tooth bone made by extracted tooth can be an acceptable method compared with xenogenic bone.
2. The stability of horizontal bone in the level 0 mm, 3 mm and 6 mm of the implant facial part was almost the between the autogenous tooth bone and the xenogenic bone at the 6 months and 12 months follow-up.
3. No matter using autogenous tooth bone or xenogenous bone, the horizontal bone loss at the first or the latter 6 months was almost the same in the level 0 mm, 3 mm and 6 mm of the implant facial part.
4. No matter what the follow-up period is and bone graft material used, the horizontal bone loss at the level of 6 mm was much less than the level of 0 mm and 3 mm in the facial of the implant.
5. Patient feel better when using the autogenous tooth bone comparing with the xenogenic bone.

## Supplementary information

**Additional file 1.** Questionnaire. Table 2 and Fig. 8. The data were based on the questionnaire about the pain, swelling and satisfaction about the surgery. And the quantitative value was based on the Visual Analog Scales (VAS 0–10).

## Data Availability

The datasets used and analysed during the current study are available from the corresponding author on reasonable request.

## Abbreviations

atuoBT: autogenous Tooth Bone
CBCT: Cone-Beam Computed Tomography
MBL: Marginal Bone Level
VAS: Visual Analog Scales
xenoB: xenogenic Bone
