# Supplementary material for: Immediate implant placement in anterior teeth with grafting material of autogenous tooth bone vs xenogenic bone
Source: BMC Oral Health. 2019 Dec 2;19:266. doi: 10.1186/s12903-019-0970-7 (PMC6889614; doi:10.1186/s12903-019-0970-7)
Supplement: Supplementary file 1 — Additional file 1. Questionnaire. Table 2 and Fig. 8. The data were based on the questionnaire about the pain, swelling and satisfaction about the surgery. And the quantitative value was based on the Visual Analog Scales (VAS 0–10). [file 12903_2019_970_MOESM1_ESM.docx]

Questionnaire of pain, swelling and the satisfaction of the surgery

Name: date:

All the answer is based on the Visual Analog Scales (VAS 0-10), 0 is none and 10 is very well.

1. How pain did you feel about the surgery?

| 0 | 1 | 2 | 3 | 4 | 5 | 6 | 7 | 8 | 9 | 10 |
| --- | --- | --- | --- | --- | --- | --- | --- | --- | --- | --- |
|  |  |  |  |  |  |  |  |  |  |  |

2. How much did you swelling during this one week?

| 0 | 1 | 2 | 3 | 4 | 5 | 6 | 7 | 8 | 9 | 10 |
| --- | --- | --- | --- | --- | --- | --- | --- | --- | --- | --- |
|  |  |  |  |  |  |  |  |  |  |  |

3. How did you satisfied with the surgery?

| 0 | 1 | 2 | 3 | 4 | 5 | 6 | 7 | 8 | 9 | 10 |
| --- | --- | --- | --- | --- | --- | --- | --- | --- | --- | --- |
|  |  |  |  |  |  |  |  |  |  |  |
